# Improvements in mentalization predict improvements in interpersonal distress in patients with mental disorders

**DOI:** 10.1002/jclp.22673

**Published:** 2018-07-11

**Authors:** Markus C. Hayden, Pia K. Müllauer, Richard Gaugeler, Birgit Senft, Sylke Andreas

**Affiliations:** ^1^ Alpen‐Adria‐Universität Klagenfurt Klagenfurt Austria; ^2^ Öffentliches Krankenhaus Waiern Feldkirchen i. K. Austria; ^3^ Reha‐Klinik für Seelische Gesundheit und Prävention Klagenfurt Austria; ^4^ Universität Witten/Herdecke Witten Germany

**Keywords:** adult attachment, interpersonal problems, mentalization, mental disorders, symptom severity

## Abstract

**Objectives:**

Associations between interpersonal problems and mentalization have rarely been investigated. In this study, we explored patterns of interpersonal problems, mentalization, symptom severity, and attachment during inpatient treatment and at follow‐up. Additionally, we investigated whether mentalization predicts a decrease in interpersonal distress.

**Method:**

We analyzed time‐series data from patients with mental disorders. Data were collected at the beginning and at the end of inpatient treatment, and approximately 6 months after discharge from hospital.

**Results:**

Patterns of correlations were stable from admission to the hospital until follow‐up. Treatment significantly increased the levels of mentalization and decreased the levels of interpersonal problems and symptom severity, whereas attachment was only partially targeted. Improvements in mentalization significantly predicted reduction in interpersonal distress at each point in time.

**Conclusion:**

Results revealed characteristic patterns of interpersonal problems, mentalization, symptom severity, and attachment. Mentalization was found to play a key role in the reduction of interpersonal distress.

## 1 INTRODUCTION

In the study of the preservation and regeneration of mental health, the concept of interpersonal problems is of significant importance. Interpersonal problems refer to recurrent patterns of difficulties that individuals experience when they interact with others (Horowitz, Dryer, & Krasnoperova, 1997). These difficulties can lead to psychic strain and, beyond that, to mental disorders (Puschner, Bauer, Horowitz, & Kordy, 2005). Various studies have documented the importance of interpersonal problems in psychopathology (Hartmann, Zeeck, & Barrett, 2010; Salzer, Pincus, Winkelbach, Leichsenring, & Leibing, 2011). Interpersonal problems are common reasons for people to seek psychotherapy and are frequently mentioned at the beginning of treatment (Locke, 2005; Puschner et al., 2005). In addition, they predict response rates and treatment outcome in psychotherapy (Newman, Jacobson, Erickson, & Fisher, 2017; Renner et al., 2012). Unfortunately, efforts to reduce interpersonal problems may require sophisticated strategies because interpersonal patterns are difficult to change (Fjeldstad, Høglend, & Lorentzen, 2017; Liebherz & Rabung, 2014; Maling, Gurtman, & Howard, 1995).

In the long history of interpersonal research, scientists have investigated a variety of concepts that are associated with interpersonal problems (Arcelus, Haslam, Farrow, & Meyer, 2013; Classen, Field, Koopman, Nevill‐Manning, & Spiegel, 2001; Hayden, Müllauer, & Andreas, 2017; Puschner et al., 2005). However, the association of interpersonal problems with the concept of mentalization (Fonagy & Target, 2006) has rarely been studied. Mentalization—or reflective functioning as operationalization of mentalization—refers to the ability to understand and interpret human behavior in terms of underlying mental states in oneself and in others. These mental states include feelings, beliefs, emotions, needs, desires, purposes, and goals (Fonagy & Target, 2006). The ability to mentalize plays a key function in interpersonal behavior for several reasons. For example, it enables humans to perceive and reflect on action and behavior. Therefore, it is vital for a differentiated understanding of human behavior (Slade, 2005). Additionally, it is of great importance in the regulation of interpersonal relationships (Fonagy & Bateman, 2006). Research has demonstrated the importance of mentalization in different areas of psychopathology (Katznelson, 2014). Disorders that include a pathology of the self, such as borderline personality disorder, are particularly closely linked to impairments in mentalization (Fonagy & Luyten, 2009). Furthermore, it is important to consider that these impairments in the ability to mentalize are associated with insecure attachment (Fonagy & Bateman, 2006; Fonagy, Steele, Steele, Moran, & Higgitt, 1991; Taubner et al., 2013), which in turn is linked to greater amounts of interpersonal problems (Hayden et al., 2017). In psychotherapy research, mentalization‐based interventions have proven to be beneficial in the treatment of borderline personality disorder (Bateman & Fonagy, 2008) and several other mental disorders (Choi‐Kain & Gunderson, 2008).

Given the importance of mentalization in psychological wellbeing and its fundamental impact on interpersonal functioning, it is remarkable that associations with interpersonal problems have rarely been investigated. In a previous study (Hayden et al., submitted), the role of mentalization and other variables in the experience of interpersonal problems was explored. In that study, characteristic patterns of associations among adult attachment, mentalization, symptom severity, and interpersonal problems in patients with mental disorders could be demonstrated. However, the authors analyzed only cross‐sectional data. In the present study, we aimed to continue our efforts to investigate the role of mentalization in interpersonal perception and behavior. Therefore, we analyzed time‐series data of patients with mental disorders from the beginning of inpatient treatment to follow‐up. First, we explored which variables improved during inpatient treatment for mental disorders. We were particularly interested in possible improvements (i.e., reduction) in interpersonal distress. Second, we investigated which variables predicted decrease in interpersonal problems over the course of therapy and at follow‐up. We hypothesized the following: Treatment would improve mental stability in terms of a reduction in interpersonal problems, attachment anxiety, attachment avoidance, and symptom severity. Levels of mentalization would increase over the course of inpatient treatment. Improvements during treatment would be stable at follow‐up and patterns of included variables would not change over the course of treatment and at follow‐up. Finally, we hypothesized that an increase in mentalization would predict a reduction in interpersonal problems

## 2 METHODS AND DESIGN

### 2.1 Procedure

Data were assessed in two Austrian medical centers for the rehabilitation of patients with mental disorders. All participants were patients of either the Public Hospital Waiern (*Öffentliches Krankenhaus Waiern*), Feldkirchen i. K., Austria, or the Rehabilitation Center Klagenfurt (*Reha‐Klinik für Seelische Gesundheit und Prävention*), Klagenfurt a. W., Austria. Both clinics use different therapeutic concepts that do not focus on a specific psychotherapy approach. The treatment plans of both medical centers include inter alia, individual and group psychotherapy, medical specialist care, psychoeducation, occupational therapy, and physical education.

All participants were between 18 and 65 years of age. Reasons for exclusion were insufficient ability to understand and/or speak German, acute psychotic or manic episode, dementia, and/or cognitive impairment. All patients who were appropriate for the study were asked to participate at the beginning of treatment. Participation was voluntary, and patients were assured that neither refusal nor posterior withdrawal was associated with any negative consequences for inpatient treatment or ambulatory after‐care. All participants signed informed consent for study participation, which included without limitation, information about the rights of the participants as well as data privacy. The study was approved by the ethical commission of the federal state of Carinthia, Austria (*Ethikkommission des Landes Kärnten*).

For the study, we used a quasi‐experimental design with time‐series data that were assessed at the beginning of inpatient therapy (T_0_), at the end of inpatient therapy (T_1_), and approximately 6 months after discharge from the clinic (T_2_).

### 2.2 Questionnaires

We used the German version of the "Inventory of Interpersonal Problems—Short Circumplex" (IIP‐32; Thomas, Brähler, & Strauß, 2011) for the assessment of interpersonal problems because it is an economic instrument that is largely comparable to the long version in terms of both reliability and validity. For the analyses, the global score that represents the amount of distress experienced in interpersonal contact was used.

The overall score of the 15‐item "Mentalization Questionnaire" (MZQ; Hausberg et al., 2012) was used to assess the ability to mentalize. This instrument proved to be both reliable and valid for the assessment and is much more economical than interview measurements.

Adult attachment was measured with the "Experiences in Close Relationship Scale (ECR)—short form" (Wei, Russell, Mallinckrodt, & Vogel, 2007). The 12‐item short version of the original ECR (Brennan, Clark, & Shaver, 1998) is a reliable, valid, and economical instrument for the assessment of adult attachment in the two‐dimensional model of attachment anxiety and attachment avoidance.

Symptom severity was assessed with the "Global Severity Index" (GSI) of the 18‐item "BSI‐18" (Spitzer et al., 2011). This short version of the *Brief Symptom Inventory (BSI)* (Franke, 2000) proved to be a reliable and valid measurement of symptom severity.

### 2.3 Data analysis

The data were analyzed using *IBM SPSS Statistics* version 25. For the analyses of differences between the two samples, we used chi‐square tests and independent samples *t*‐tests. For the *t*‐tests, homoscedasticity was verified by Levene's test for equality of variances (Levene, 1960). To evaluate whether patterns of mentalization, interpersonal problems, attachment, and symptom severity were stable over time, we analyzed the variables using Pearson product‐moment correlations for each point in time. For the analysis of changes within the included variables over time, we used repeated‐measures one‐way analyses of variances (ANOVAs). All variables that were analyzed by the ANOVAs were checked for sphericity using Mauchly's sphericity test.

For the analysis of the second research question, we used linear regression models. For interpersonal problems, symptom severity, and both attachment dimensions, we calculated the improvement during treatment by subtracting the value of T_1_ from the value of T_0_. Likewise, we calculated the improvement at follow‐up by subtracting the value of T_2_ from the value of T_1_. Since mentalization is described inversely, an improvement is represented as an increase in MZQ‐scores. Therefore, we calculated the development for this variable vice versa and subtracted the value of T_0_ from the value of T_1_ and likewise the value of T_1_ from the value of T_2_.

## 3 RESULTS

### 3.1 Sample

Fifty‐seven patients of the Hospital Waiern and 32 patients of the Rehabilitation Center Klagenfurt were willing to take part in the study. The description of the sample is displayed in Table 1. The vast majority of patients had ICD‐10 mood disorders (F3) or neurotic, stress‐related, and somatoform disorders (F4) (World Health Organization, 1992) as main diagnoses.

**Table 1:** Sample description

		Hospital Waiern	Rehabilitation Center	Total
***n***		57	32	**89**
**Age**		M 44.2 (SD 9.92)	M 43.7 (SD 9.70)	**M 44.0 (SD 9.79)**
**Sex**	Male	29	13	**42 (47.2%)**
	Female	28	19	**47 (52.8%)**
**Education**	Elementary	0	1	**1 (1.1%)**
	Main school	12	13	**25 (28.1%)**
	Professional school	8	7	**15 (16.9%)**
	High school	13	4	**17 (19.1%)**
	University	11	3	**14 (15.7%)**
	Other	13	4	**17 (19.1%)**
**Civil status**	Single	11	15	**26 (29.2%)**
	Living in partnership or married	33	11	**44 (49.5%)**
	Divorced or widowed	13	6	**19 (21.3%)**
**Children**	Yes	36	20	**56 (62.9%)**
	No	21	12	**33 (37.1%)**
**Diagnoses**	F20 Schizophrenia	0	1	**1 (1.1%)**
	F25 Schizoaffective disorders	0	1	**1 (1.1%)**
	F29 Unspecified nonorganic psychosis	0	1	**1 (1.1%)**
	F31 Bipolar affective disorder	5	2	**7 (7.8%)**
	F32 Depressive episode	23	5	**28 (31.6%)**
	F33 Recurrent depressive disorder	18	8	**26 (29.3%)**
	F41 Other anxiety disorders	7	2	**9 (10.1%)**
	F43 Reaction to severe stress, and adjustment disorders	1	12	**13 (14.6%)**
	F45 Somatoform disorders	2	0	**2 (2.2%)**
	Other	1	0	**1 (1.1%)**

#### 3.1.1 Differences between the subsamples

Regarding sociodemographic parameters, there were no statistically significant differences between the two subsamples in sex, age, level of education, current employment, or the number of children. The only significant difference that could be detected was related to civil status (*χ^2^*_(2)_ = 7.786, *P* = 0.020, *V*_Cramer_ = 0.296). For a closer analysis of differences, we calculated independent samples *t*‐tests for all included variables. Levene's test did not find any inhomogeneous variances. Data revealed that only three variables differed significantly. Each of these variables differed at one point in time only. Patients of the Rehabilitation Center Klagenfurt experienced significantly more attachment anxiety at T_1_ (*t*_(70)_ = –2.016, *P *= 0.048) and had more interpersonal problems at T_2_ (*t*_(69)_ = –2.543, *P *= 0.013) than patients of the Hospital Waiern. However, they showed significantly lower scores of mentalization at follow‐up (*t*_(69)_ = 2.595, *P *= 0.012). Effect sizes, as described by Cohen's *d*, ranged from *d* = 0.49 for attachment anxiety to *d* = –0.62 for mentalization. The differences between the samples of the two medical centers indicated only small to medium effects (Cohen, 1988) and were not consistent over time. Hence, we concluded that there were no substantial differences between the two samples.

#### 3.1.2 Dropout

Of the 89 patients who participated at T_0_, *n* = 3 (3.4%) dropped out during the inpatient treatment. One assessment was cancelled by the examiner in charge due to an acute emotional crisis of the participant, whereas two other patients had no further interest in the study. Another *n* = 15 (16.9%) participants dropped out at T_2_. Reasons for dropping out were severe illness (*n* = 1), death (*n* = 1), and no further interest in the study (*n* = 2). Nine patients could not be reached at follow‐up, and one assessment was cancelled by the examiner in charge due to the labile mental situation of the participant. One participant did not specify his withdrawal. Seventy‐one patients (79.8%) finished the data collection; therefore, the overall dropout rate over the course of the study was acceptable since rates greater than 30% are not uncommon in scientific studies (National Research Council, 2010).

There were some statistically significant baseline differences between the dropouts and nondropouts. Participants who dropped out of the study had higher levels of interpersonal distress (*d* = 0.664) and symptom severity (*d* = 0.746) but lower scores of mentalization (*d* = –0.548) at the beginning of treatment.

### 3.2 Stability of intercorrelations between the included variables

Pearson product‐moment correlations of the included variables at the beginning of treatment revealed significant positive correlations between attachment anxiety, attachment avoidance, interpersonal distress, and symptom severity, ranging from *r *= 0.30 to *r* = 0.60. Mentalization was significantly negatively correlated with all other variables. Correlation coefficients ranged from *r *= –0.38 to *r* = –0.75. Attachment anxiety and attachment avoidance were not significantly intercorrelated (see Hayden et al., submitted, for more details). The results for T_1_ and T_2_ are displayed in Table 2. Since values for GSI were not normally distributed, we used the decadic logarithm of the results for this variable at both T_1_ and T_2_. As shown, attachment, interpersonal problems, and symptom severity were significantly positively correlated with each other at both T_1_ and T_2_. Correlation coefficients ranged from *r* = 0.34 to *r* = 0.76. Surprisingly, the two dimensions of adult attachment were also significantly correlated at both times of measurement (*r* = 0.39 for T_1_ and *r* = 0.36 for T_2_). As expected, mentalization turned out to be negatively correlated with all other variables at both T_1_ (*r* = –0.42 to *r* = –0.74) and T_2_ (*r* = –0.57 to *r* = –0.78). Except for the correlation between attachment anxiety and attachment avoidance, all correlations were noticeably higher at T_2_.

**Table 2:** Correlation matrix of included variables

T_1_	Attachment anxiety	Attachment avoidance	MZQ total score	IIP total score	GSI
**Attachment anxiety**	—	0.386**	–0.464***	0.490***	0.443***
		*n* = 72	*n* = 64	*n* = 71	*n* = 66
**Attachment avoidance**		—	–0.418**	0.343**	0.425***
			*n* = 64	*n* = 71	*n* = 66
**MZQ total score**			—	–0.737***	–0.663***
				*n* = 66	*n* = 62
**IIP total score**				—	0.668***
					*n* = 68
**GSI**					—

T_2_	Attachment anxiety	Attachment avoidance	MZQ total score	IIP total score	GSI
**Attachment anxiety**	**—**	0.362**	–0.689***	0.572***	0.729***
		*n* = 68	*n* = 68	*n* = 68	*n* = 64
**Attachment avoidance**		**—**	–0.573***	0.473***	0.471***
			*n* = 68	*n* = 68	*n* = 64
**MZQ total score**			**—**	–0.788***	–0.777***
				*n* = 71	*n* = 67
**IIP total score**				**—**	0.760***
					*n* = 67
**GSI**					**—**

GSI, Global severity index; IIP, Inventory of interpersonal problems; MZQ, Mentalization questionnaire;

**P < 0.01; ***P < 0.001 (*P*‐values are two tailed).

Differences in *n* are caused by missing data.

### 3.3 Changes over the course of treatment and posttreatment

In the next step, we used repeated‐measures ANOVAs to examine changes in the different variables over time. Violations of sphericity were detected in the analyses of attachment anxiety, mentalization, and interpersonal problems. In all cases, violations turned out to be on a level of *ε* > 0.75. Based on the recommendations of Girden (1992), we adjusted the analyses using the Huynh–Feldt correction (Huynh & Feldt, 1976). Results of the analyses are displayed in Table 3. As shown, attachment anxiety did not reveal any significant changes (*F*(1.864, 106.235) = 1.162, *P* = 0.314). All other variables improved significantly over time. Effect sizes, as described by partial eta‐squared, ranged from *η* ^2^ = 0.080 for attachment avoidance to *η* ^2^ = 0.290 for symptom severity. Post hoc tests with the Bonferroni procedure revealed significant effects between T_0_ and T_1_ for each variable except for attachment avoidance. Mentalization, interpersonal problems, attachment avoidance, and symptom severity differed significantly between T_0_ and T_2_. Between the discharge from the clinic and the follow‐up assessment, only mentalization differed significantly.

**Table 3:** Repeated‐measure ANOVA results of included variables

	*F*‐statistics	Partial *η* ^2^
**Attachment anxiety**	*F*(1.864, 106.235) = 1.162	0.030 n.s.
**Attachment avoidance**	*F*(2, 114) = 4.988	**0.080** **
**Mentalization**	*F*(1.625, 94.277) = 13.364	**0.187** ***
**Interpersonal problems**	*F*(1.724, 106.858) = 6.512	**0.095** **
**Symptom severity**	*F*(2, 114) = 23.303	**0.290** ***

**P < 0.01; ***P < 0.001; n.s., not significant.

### 3.4 Prediction of relief in interpersonal distress

The most important step in our study was the analysis of the improvement of interpersonal problems. We investigated whether the decrease in interpersonal distress is predicted by the improvements in one or more of the other variables. Rates of improvement were computed as described above. We calculated two linear regression analyses with stepwise procedures relying on *F*‐probability criteria of 0.05 to enter and 0.10 to remove variables. Two significant models were obtained: one for the duration of treatment (*R* ^2^ = 0.241, *F*(2, 54) = 8.580, *P* = 0.001) and one for the duration between the end of the treatment and the follow‐up assessment (*R* ^2^ = 0.234, *F*(1, 52) = 15.895, *P* < 0.001). The results for the analyses are displayed in Table 4. During treatment, the improvement in mentalization (standardized *β* = 0.324, *P *= 0.013) and in symptom severity (standardized *β* = 0.276, *P *= 0.033) were significant predictors of the decrease in interpersonal problems. Posttreatment, the reduction in interpersonal problems was only significantly predicted by the increase in mentalization (standardized *β* = 0.484, *P *< 0.001).

**Table 4:** Results from the linear regression models that predict improvement in interpersonal problems

(1) Improvement in interpersonal distress during treatment (T_0_ – T_1_)		
Predictor variable	Standardized *β*	Change in *R^2^*
Improvement in mentalization	0.324*	0.174
Improvement in symptom severity	0.276*	0.067

(2) Improvement in interpersonal distress at follow‐up (T_1_ – T_2_)		
Predictor variable	Standardized *β*	Change in *R^2^*
Improvement in mentalization	0.484***	0.234

(3) Total improvement in interpersonal problems (T_0_ – T_2_)		
Predictor variable	Standardized *β*	Change in *R^2^*
Improvement in attachment anxiety	0.450***	0.471
Improvement in mentalization	0.406***	0.110

*P < 0.05; **P < 0.01; ***P < 0.001

Since the increase in mentalization was a significant predictor of the decrease in interpersonal problems at both points in time, we calculated a third model. We investigated whether the effect was stable over the entire time period from the admission to the hospital until follow‐up. Improvement of variables between T_0_ and T_2_ was calculated, and a stepwise multiple regression analysis was utilized as previously described. The results of the analysis are presented in Table 4. As shown, the model was stable (*R* ^2^ = 0.580, *F*(2, 54) = 37.227, *P* < 0.001), and the increase in mentalization significantly predicted the reduction of IIP‐scores (standardized *β* = 0.406, *P *< 0.001). However, the decrease in attachment anxiety was also a significant predictor (standardized *β* = 0.450, *P *< 0.001).

## 4 DISCUSSION

In the present study, we investigated the role of mentalization in the improvement of interpersonal problems over the course of inpatient psychotherapy and follow‐up. To obtain comprehensive and more detailed results, we included symptom severity and adult attachment in our analyses. We investigated patients with mental disorders at the beginning and at the end of treatment as well as approximately 6 months after the discharge from the hospital.

Bivariate correlations found characteristic associations between all the included variables at each assessment date. Attachment anxiety, attachment avoidance, interpersonal problems, and symptom severity were positively correlated with medium and large effects. The strongest associations were the ones between symptom severity and interpersonal problems. The weakest associations could be detected between attachment avoidance and symptom severity and between attachment avoidance and interpersonal problems. These results are consistent with previous studies (e.g., Berry, Barrowclough, & Wearden, 2008; Haggerty, Hilsenroth, & Vala‐Stewart, 2009; Lorentzen, Fjeldstad, Ruud, & Høglend, 2015) and confirm the assumption of specific associations among attachment, interpersonal behavior, and the occurrence of mental symptoms. Consistent with our presumptions, mentalization was negatively correlated with all other variables. Correlation coefficients ranged from *r* = –0.42 to *r* = –0.74 at T_1_ and from *r* = –0.57 to *r* = –0.78 at T_2_. Effect sizes were moderate for the association between attachment and mentalization at T_1_ and large for all of the other associations. These findings verify the results of previous analyses (Hayden et al., submitted) and support the hypothesis that patients who experience more distress in interpersonal contact face more attachment insecurities and experience greater amounts of symptom severity. This appears to be associated with greater difficulties to mentalize, as other studies have suggested (Bateman & Fonagy, 2009; Hausberg et al., 2012; Taubner et al., 2013). In the current study, it is important to emphasize that all associations were noticeably stronger at the termination of treatment and at follow‐up. Therefore, the patterns of associations between the variables intensified over the course of treatment and further intensified at follow‐up. This finding supports the idea that characteristic intraindividual styles of attachment, interpersonal behavior, and mentalization are associated with psychopathology.

Surprisingly, the two dimensions of adult attachment were significantly correlated at both T_1_ and T_2_. Following Cohen (1988), effect sizes can be labeled moderate for both points in time. This association was not found in previous studies (Hayden et al., submitted) and is in contrast to the theory of the ECR (Brennan et al., 1998; Wei et al., 2007). However, other researchers have found similar results and have noted the plausibility of intercorrelation between the two dimensions (e.g., Conradi, Gerlsma, Duijn, & Jonge, 2006; Fraley, Heffernan, Vicary, & Brumbaugh, 2011). Even if attachment anxiety and attachment avoidance are conceptually distinct and clearly separable, statistical independence will not always be measurable (Fraley et al., 2011). With regard to our data, the increase in intercorrelation may be caused by the changes in attachment avoidance.

Univariate ANOVAs revealed decreases in both symptom severity and interpersonal problems over the course of therapy as well as an increase in mentalization. Regarding interpersonal problems and symptom severity, significant changes between T_0_ and T_2_ indicate that the improvements during treatment remained stable after discharge from the hospital. Mentalization even increased significantly between discharge from the hospital and follow‐up. Attachment anxiety was stable during the entire process, which suggests that therapy did not significantly affect this variable. Since other studies have provided evidence for changes in attachment anxiety during treatment (Taylor, Rietzschel, Danquah, & Berry, 2015), an explanation may be that the therapy approaches that were used in the clinics focused more on other spheres of human perception and behavior. The results for attachment avoidance revealed significant differences between T_0_ and T_2_. However, the differences between the admission to inpatient treatment and the end of therapy did not reach statistical significance, although they approximated the threshold of .05 (*P *= .051). This finding indicates that the promising trends during therapy continued after the end of inpatient therapy and produced significant results with a medium effect. However, it is important to consider that due to the dropouts and some missing data in the questionnaires, the power of the analysis was not optimal. Considering the *P*‐value of 0.051, we assume that an adequate sample size would have led to significant results in this variable. Since patters of adult attachment are evaluated as relatively stable (Ravitz, Maunder, Hunter, Sthankiya, & Lancee, 2010), the overall decrease in attachment avoidance appears surprising. However, research has demonstrated that changes during psychotherapy are at least plausible (Taylor et al., 2015).

Regression analyses indicated that mentalization is of great importance in the recovery of mental health. All three of the models that we calculated revealed that improvements in mentalization significantly predict improvements in interpersonal problems. The first model explained 24.1% of the variance in the decrease in interpersonal problems over the course of treatment. The second model explained 23.4% of the variance in the posttreatment improvement. The common predictor of both models was mentalization. Since levels of interpersonal problems differed significantly between T_0_ and T_2_, we calculated a third model for the whole time period. Again, the increase in mentalization turned out to be a significant predictor. In line with related research (Bateman & Fonagy, 2009; De Meulemeester, Lowyck, Vermote, Verhaest, & Luyten, 2017), our data suggest that an increase in mentalization accompanies a reduction in interpersonal distress. Our findings underline the importance of mentalization in the rehabilitation of mental disorders and the preservation of psychological wellbeing. Since interpersonal problems are common reasons for people to seek psychotherapy (Locke, 2005), the relief of interpersonal distress is a topic of great importance in psychotherapy research. In terms of therapy strategies for this issue, an advancement of mentalization appears to be advisable regardless of the therapy approach that is applied. In particular, this advice addresses the fact that attachment anxiety could not be targeted with the therapy strategies used at the clinics, whereas mentalization was significantly increased during and after treatment.

## 5 LIMITATIONS

There are some limitations to our study that must be mentioned. First, there were a few significant differences between patients who completed the study and those who dropped out. The dropouts had lower scores of mentalization and were more burdened by symptom severity and interpersonal problems. Therefore, the findings should be generalized with caution. It is important to consider that the results may be valid only for persons who are not severely burdened by mental strain. Patients with greater psychopathology may reveal different results. Nevertheless, we have to point out that most of the dropouts were caused because patients could not be reached. Second, due to the dropouts and some missing data in the questionnaires, the analyses of improvement in attachment avoidance were not optimally strong with regard to statistical power. This may have had an impact in the post hoc analysis of the conducted ANOVA; a larger sample may have led to a significant difference between T_0_ and T_1_. Third, the treatment that patients received at the two medical centers did not focus on one or more of our key variables (mentalization, interpersonal problems, or attachment). In the present study, this approach was chosen deliberately. Nevertheless, it is important for further studies, to test the proposed model in experimental designs that use specific therapeutic approaches, such as mentalization‐based treatment (Bateman & Fonagy, 2004) or interpersonal psychotherapy (Markowitz & Weissman, 2004).

## 6 CONCLUSION

Our results revealed consistent patterns of intercorrelations between interpersonal problems, mentalization, adult attachment, and symptom severity in patients with mental disorders. Patients with higher amounts of interpersonal distress had more difficulties in mentalizing and were more burdened by symptom severity and attachment insecurities. These patterns remained stable over the course of inpatient treatment and at follow‐up. After therapy, levels of interpersonal problems, mentalization, and symptom severity had significantly improved. Interpersonal problems and symptom severity remained stable at follow‐up, whereas mentalization further increased. Attachment anxiety could not be targeted. Attachment avoidance significantly decreased between the admission to hospital and follow‐up. During treatment, the reduction was not significant but revealed a positive trend. Linear regression analyses revealed that improvements in interpersonal problems were predicted by improvements in mentalization at each time interval. The results of the study underline the importance of mentalization in the rehabilitation from mental disorders and especially in the reduction of interpersonal distress. In light of the findings, we recommend strategies to promote mentalization in the therapy of interpersonal problems regardless of the therapy approach that is applied.
